# *Pseudomonas xanthomarina* causing ventriculoperitoneal shunt infection: a case report

**DOI:** 10.3389/fmed.2026.1706619

**Published:** 2026-04-24

**Authors:** Chenglin Wang, Ning Su, Shuai Dang

**Affiliations:** 1 Trauma and Neurocritical Care Unit, Nanyang Central Hospital, Nanyang, China; 2 Department of Neurology, Nanyang Central Hospital, Nanyang, China

**Keywords:** case report, central nervous system, *Pseudomonas xanthomarina*, retrograde infection, ventriculoperitoneal shunt

## Abstract

Infection following ventriculoperitoneal shunt surgery is a serious complication. Among severe infections caused by *Pseudomonas*, *non-Pseudomonas aeruginosa* infections are relatively rare, and there are currently no case reports of central nervous system infections caused by *Pseudomonas xanthomarina*. Here, we present the first case of retrograde central nervous system infection by *Pseudomonas xanthomarina* following ventriculoperitoneal shunt placement.

## 1 Introduction

Ventriculoperitoneal (VP) shunt surgery is the most commonly used procedure for treating hydrocephalus in clinical practice (1). With advancements in surgical techniques and improvements in implant materials, the incidence of complications has decreased compared to earlier periods. However, infection remains the most serious complication, occurring in approximately 10% of cases and substantially increasing both mortality and economic burden (2–5).

Skin flora is the main source of infection (6). Recent studies confirm *Staphylococcus epidermidis*, *Staphylococcus aureus*, and Gram-negative rods are the most common pathogens in cerebrospinal fluid (CSF) shunt infections, with *S. epidermidis* remaining the most frequently isolated organism (7–11). In severe infections caused by *Pseudomonas* species, *non-Pseudomonas aeruginosa* infections are relatively rare. *Pseudomonas xanthomarina* is a Gram-negative bacterium of the *Pseudomonas* genus. It was originally isolated from the sea and was first described by Romanenko et al. (12). It is an aerobic, denitrifying, psychrotolerant, rod-shaped cell with yellow-orange pigmentation, a lack of fluorescence, and motility conferred by a single polar flagellum. Growth occurs in 0–8% NaCl and at temperatures between 4 and 37 °C, but not in 10% NaCl or at 40–41 °C. The strain is susceptible to ampicillin (10 μg), gentamicin (10 μg), kanamycin (30 μg), carbenicillin (25 μg), lincomycin (15 μg), oleandomycin (15 μg), polymyxin (300 U), streptomycin (30 μg), tetracycline (30 μg), and neomycin (15 μg), while demonstrating resistance to benzylpenicillin (10 U).

To date, there have been no cases of central nervous system (CNS) infection caused by *Pseudomonas xanthomarina*. Our case report first presents a rare early-onset retrograde infection following VP shunt, which was attributed to *Pseudomonas xanthomarina*.

## 2 Case report

A 61-year-old female presented in a comatose state due to a subarachnoid and intraventricular hemorrhages caused by a ruptured aneurysm in the communicating segment of the internal carotid artery. Detailed demographic data are presented in Table 1. Aneurysm clipping and decompressive craniectomy were immediately conducted at the local hospital. One month later, a VP shunt was performed for obvious hydrocephalus on the premise that C-reactive protein (CRP), white blood cells (WBC), and CSF indicators were normal. After the operation, the patient can open her eyes independently without any consciousness. However, three days later, intermittent fever began to occur, with body temperature fluctuating between 37.8 °C and 38.5 °C, and the patient’s mental state deteriorated, with a notable absence of eye-opening movements. Additionally, blood pressure also dropped and a small dose of vasoactive drugs was administered to maintain it. Therefore, the patient was transferred to our hospital for further treatment.

**Table 1:** Demographics of the patient.

Demographics	Results
Age	61 years old
Gender	Female
Ethnicity	Han Chinese
Marital status	Married
Occupation	Farmer
Residence	Village
Height	160 cm
Weight	75 kg
BMI	29.3 kg/m^2^
Smoking history	None
Alcohol consumption	None
Menstrual/Reproductive history	Postmenopausal, no abnormal history
Allergy history	None
Comorbidities	Hypertension
Householdremedy	Valsartan capsules
Recent hospitalization	Aneurysm clipping and decompressive craniectomy (1 month prior)
Psychosocial history	No special findings

BMI, body mass index.

The patient was comatose on admission, with a Glasgow Coma Scale (GCS) score of 2 + T. Both pupils were 3 mm in diameter, round, and non-reactive to light. No spontaneous movement occurred in the extremities, and no motor response to painful stimuli was present. Muscle tone was decreased in all four limbs, and plantar responses were extensor bilaterally, indicating a positive Babinski sign. Meningeal signs, including nuchal rigidity, Kernig’s sign, and Brudzinski’s sign, were absent. Abdominal examination revealed only distension and slightly diminished bowel sounds, with no other abnormalities.

**Figure 1:**
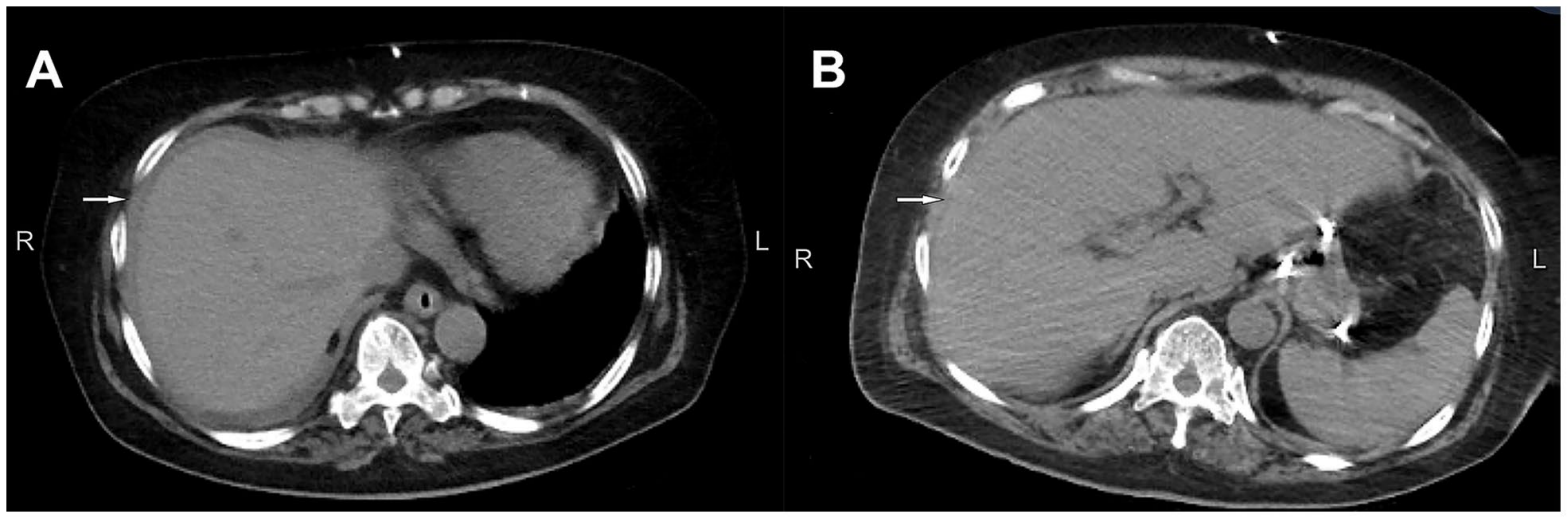
Abdominal CT scan. **(A)** CT on admission indicated a small amount of fluid around the liver. **(B)** On the seventh day of admission, CT showed that the fluid accumulation around the liver had disappeared. CT, computed tomography.

**Figure 2:**
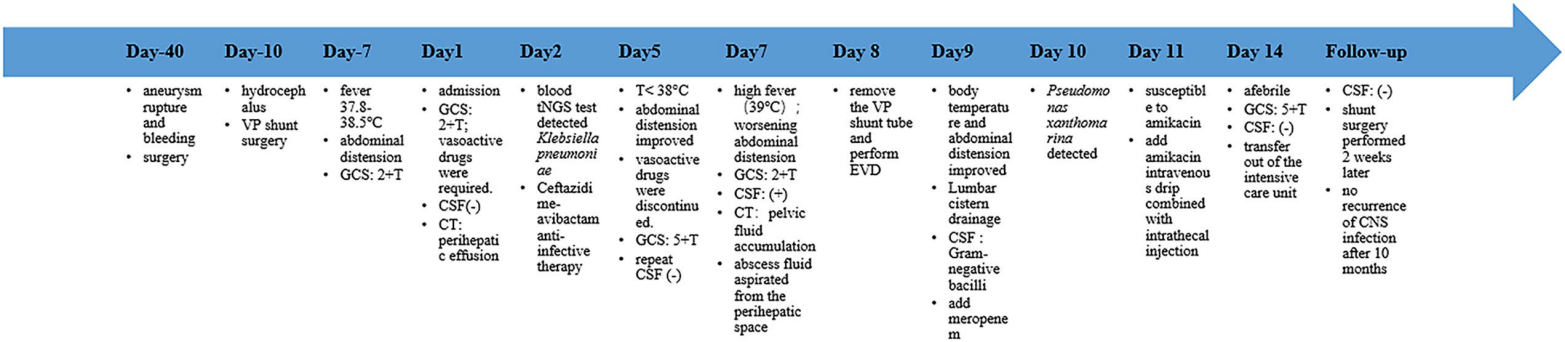
Timeline of the patient’s clinical course. The timeline illustrates the sequence of key events from symptom onset to follow-up. Day 1 is defined as the date of admission. VP, ventriculoperitoneal; GCS, Glasgow Coma Scale; CSF, cerebrospinal fluid; CT, computed tomography; T, temperature; EVD, external ventricular drainage; CNS, central nervous system.

Meanwhile, we immediately conducted a detailed investigation into the source of infection and sent blood, CSF, sputum, and urine for bacterial culture tests, as well as targeted high-throughput sequencing (tNGS) in blood and CSF. The initial CSF count yielded WBC 214 × 10^^6^/L, glucose 2.42 mmol/L, and protein 1,592.6 mg/L. No pathogen was detected in CSF tNGS and bacterial culture, while *Klebsiella pneumoniae* was detected in blood tNGS, with a sequence number of 3. The blood routine test indicated that the total WBC count was normal and CRP was 159.98 mg/L. The corresponding laboratory results are summarized in Table 2. Computed tomography (CT) revealed hydrocephalus, mild pulmonary infection, intestinal distension, and a small amount of fluid around the liver (Figure 1A). After consultation in the general surgery department, it was considered that there were no indications for a surgical operation. Given the poor clinical outcomes of meropenem and vancomycin administered by the local hospital, we switched to Ceftazidime and Avibactam. Three days later, the re-examination of the lumbar puncture CSF index showed: WBC 9 × 10^^6^/L, glucose 2.65 mmol/L, and protein 370.5 mg/L (Table 2). The result of CSF bacterial culture is negative. The patient’s fever frequency decreased compared to before, and the highest body temperature did not exceed 38 °C. Vasoactive drugs were quickly discontinued, and the patient’s consciousness returned to that before the operation. The patient opened her eyes spontaneously but remained unaware, with a GCS score of 5 + T. Other neurological examination findings showed no significant changes. Abdominal distension had decreased relative to its prior state. But unfortunately, the patient suddenly developed a high fever with a body temperature of 39 °C, and abdominal distension worsened on the seventh day of admission. GCS score subsequently declined to 2 + T. Re-examination of blood routine showed that the total WBC count was 15.17 × 10^^9^/L, CRP was 239.93 mg/L. The appearance of the CSF was re-examined and it was light yellow, clear fluid. CSF count yielded WBC 1,147 × 10^^6^/L, glucose 0.86 mmol/L, and protein 1,509.7 mg/L. No RBC were found (Table 2). CT showed that the fluid collection around the liver disappeared, and a small amount of pelvic fluid collection was newly added (Figure 1B). Under the guidance of color Doppler ultrasound, a small amount of yellow turbid fluid was punctured around the liver and sent for bacterial culture. Considering the CNS infection combined with abdominal infection, the removal of the VP shunt and external ventricular drainage were immediately performed (on the second day, it was changed to continuous lumbar drainage of cerebrospinal fluid) the next day. The body temperature and abdominal distension improved significantly after the operation. The infectious organism of CSF, ventricular end and abdominal end of the shunt tube, and abdominal pus was subsequently identified as *Pseudomonas xanthomarina*, whose antibiotic resistance profile is shown in Table 3. Collectively, these findings meet the diagnostic criteria for CNS infection established by the CDC/NHSN (13). We started treatment with meropenem (MEM;6 g/d; the infusion time per group is more than 3 h) and amikacin (AMK;15 mg/Kg/d) intravenously and by intrathecal injection of AMK (30 mg/d). Table 4 details the specific interventions, changes, and their rationale. The body temperature and state of consciousness gradually improved; meanwhile, the CRP, PCT, WBC counts, and CSF findings were all non-specific, and multiple CSF bacterial cultures were negative. The case timeline is presented in Figure 2.

**Table 2:** Laboratory data.

Laboratory index (Unit)	Normal range of value	On admission	Day 3 after admission	Day 7 after admission
CSF indicators				
RBC (10^9^/L)	0	43	0	0
WBC (10^6^/L)	0–8	214	9	1,147
Glucose (mmol/L)	2.5–4.9	2.42	2.65	0.86
Protein (mg/L)	200–450	1,592.6	370.5	1,509.7
Bacterial culture	Negative	Negative	Negative	Positive
Blood indicators				
Total WBC count (10^9^/L)	3.50–9.50	9.21	N	15.17
Neutrophil Percentage (%)	40.0–75.0	81.1	N	86.3
CRP (mg/L)	0–10	159.98	N	239.93
IL-6 (pg/mL)	0–7	71.81	N	259.6
PCT (ng/mL)	0–0.046	0.359	N	0.423

CSF, cerebrospinal fluid; RBC, red blood cells; WBC, white blood cells; CRP, C-reactive protein; IL-6, Interleukin-6; PCT, Procalcitonin; N, not examined.

**Table 3:** Antibiotic sensitivities of *Pseudomonas xanthomarina*.

Antibiotic	MIC (ug/mL)	Susceptibility
Piperacillin	≥128	Resistant
Ceftriaxone	≥64	Resistant
Cefotaxime	≥64	Resistant
Ceftazidime	≥64	Resistant
Cefepime	≥64	Resistant
Aztreonam	=8	Sensitive
Ticarcillin/clavulanic acid‌	≥128/2	Resistant
Piperacillin/tazobactam	≥128/4	Resistant
Cefperazone-sulbactam	≥128/32	Resistant
Meropenem	≥32	Resistant
Gentamicin	≥32	Resistant
Tobramycin	≥32	Resistant
Amikacin	=16	Sensitive
Trimesulf	≥8/76	Resistant
Chloramphenicol	≥64	Resistant

MIC, minimal inhibitory concentration.

**Table 4:** Changes in the interventions with rationale.

Time point	Change	Rationale
Day 1	Meropenem and vancomycin were initiated	Clinical suspicion of CNS infection
Day 2	Ceftazidime-avibactam was added; meropenem and vancomycin were stopped	Blood tNGS detected *K. pneumoniae* and CSF tNGS was negative
Day 8	VP shunt was removed and EVD was placed	Persistent high fever; abdominal and CNS infection
Day 9	Meropenem was restarted; EVD was replaced with lumbar CSF drainage	Gram-negative bacteria were detected in CSF, shunt, and abdominal pus
Day 11	Amikacin was added as targeted therapy	Culture and susceptibility results were available

CNS, central nervous system; CSF, cerebrospinal fluid; VP, ventriculoperitoneal; EVD, external ventricular drainage.

Several challenges were encountered. First, *Pseudomonas xanthomarina* is an exceptionally rare cause of VP shunt-associated CNS infection, with no prior cases reported. Second, the patient presented with concurrent abdominal and CNS symptoms, making it difficult to determine whether the infection originated in the peritoneal cavity and migrated retrograde to the CNS, or vice versa. The temporal sequence, in which abdominal infection preceded CNS infection, supported a retrograde mechanism.

The patient was followed up by telephone for 10 months after discharge. A new shunt was placed 2 weeks following a negative CSF culture. At the final follow-up, the patient was afebrile and exhibited no symptoms of CNS infection or elevated intracranial pressure. She continued with systematic rehabilitation therapy, could open her eyes spontaneously, but remained unable to follow commands. Her limbs could be moved horizontally on the bed. The cranial and abdominal surgical incisions had healed well, without redness, swelling, exudate, or sinus tract formation. Follow-up CSF analysis revealed normal WBC counts, protein, and glucose levels. CSF bacterial cultures were negative, with no recurrence of CNS infection. The shunt was correctly positioned without displacement or obstruction, confirming the patency of the system. Treatment adherence was confirmed by direct observation and telephone follow-up with caregivers. No adverse events, including infection recurrence, shunt malfunction, surgical complications, or antibiotic intolerance, occurred during the follow-up period. The persistent neurological deficits observed are consistent with the expected sequelae of the initial aneurysm rupture and hemorrhage.

## 3 Discussion

Catheter colonization during surgery is the most common mechanism of shunt-related infection (6, 14), with most infections occurring within the first month postoperatively (15–17). In contrast, retrograde infection occurring from the distal end of the shunt is relatively rare. Later-onset infection may result from intestinal perforation caused by the distal catheter or from transluminal passage of bacteria even in the absence of perforation (6, 16, 18).

We consider that the pathogenesis in this patient involved intestinal dysfunction, which led to bacterial translocation to the abdominal end of the shunt. This process resulted in abdominal infection and, subsequently, retrograde infection of the CNS. Intestinal dysfunction secondary to long-term bed rest after coma may be the cause of the early infection in this patient. Preoperative CSF findings were negative. The results of CSF at the two times after admission were both negative, and the initial CSF manifestation was considered to be caused by paracentesis injury. After the patient’s condition aggravated, the repeat CSF analysis and the character of the abdominal paracentesis fluid strongly suggested bacterial infection. Following surgical removal of the shunt tube, symptoms improved significantly, especially abdominal signs and fever. Ultimately, the same pathogen was cultured from the ventricular and abdominal ends of the VP shunt tube, CSF, and abdominal pus, namely, *Pseudomonas xanthomarina*, which is clinically rare and most likely derived from the intestine.

Spontaneous bacterial peritonitis (SBP) is an infection of the peritoneal fluid in the absence of an obvious abdominal lesion, and the CSF within the abdominal cavity may provide the necessary medium for the occurrence of SBP (19). The traits of SBP include a high incidence of bacterial infection, and these bacteria belong to the types considered to be normal intestinal flora (19, 20). *Pseudomonas* is not a typical member of the gut microbiota, but it has strong metabolic and pathogenic potential (21). When the gut microbiota of critically ill patients is disrupted, the gut can act as a reservoir for *Pseudomonas* and transmit to the lungs and other infected sites. The intestinal mucosa, as a major local defense barrier, prevents bacteria and endotoxins in the intestine from escaping and invading distant organs and tissues. Intestinal injury may lead to changes in intestinal barrier function, thus leading to bacterial translocation (22). Although the downward flow of CSF helps prevent the spread of infection from the abdominal cavity upward, there is no barrier outside the shunt to prevent the pathogen from spreading upward (19). The polysaccharide ‘slime layer’ produced by *Pseudomonas* can impair phagocytosis of the organism (23, 24), which may play a role in its retrograde infection along the outside of the shunt to the CNS. The culture of *Pseudomonas xanthomarina* from both the cerebral ventricle end and the abdominal cavity end of the shunt tube further supports the possibility of retrograde infection from the outside of the shunt tube.

The studies by Pelegrín et al., Campbell et al., Claunch et al., and Sener et al. (7, 8, 10, 11) confirm that coagulase-negative staphylococci, particularly *Staphylococcus epidermidis*, are the most frequently isolated pathogens in VP shunt infections. A recent retrospective study by Wang et al. (14) reported that Gram-positive cocci, primarily coagulase-negative staphylococci, caused most early VP shunt infections, whereas Gram-negative bacilli predominated in late-onset cases. Retrograde infection represents the most common cause of delayed infection (14). Our patient developed an early postoperative retrograde infection with pathogens outside this typical microbial spectrum, a discrepancy we believe warrants attention.

A positive CSF bacterial culture remains the diagnostic gold standard for shunt infection following VP shunt surgery, distinguishing it from shunt obstruction, recurrent aneurysm rupture, postoperative aseptic meningitis, or simple abdominal infection. In clinical practice, however, the infections may present with subtle or nonspecific findings, often complicated by biofilm formation and delayed microbiological detection. In some cases, prolonged incubation of CSF or shunt material is required, particularly for slow-growing organisms such as *Propionibacterium acnes* (25), which poses a significant diagnostic challenge.

To date, no randomized trials have established a management gold standard for VP shunt infections. According to the 2017 IDSA guidelines, management should involve device removal, external drainage placement, intravenous antibiotic therapy, and shunt reinsertion after CSF sterilization (26). Device removal is the critical therapeutic step, as pathogens forming biofilms on prosthetic material are not eradicated by antibiotics alone.

Outcomes for carbapenem-resistant Gram-negative bacteria-related healthcare-associated ventriculitis and meningitis remain poor. In the study by Ye et al. (27), treatment was ineffective in 45.7% of cases, and 55.4% had poor prognosis (dependent daily living, vegetative state, or death).

This case report has several notable strengths. First, to our knowledge, this is the first reported case of CNS infection caused by *Pseudomonas xanthomarina* following VP shunt surgery, representing a valuable addition to the literature. Second, the case provides a clear temporal sequence supporting early retrograde infection from the peritoneal cavity to the CNS. Third, the detailed microbiological analysis and successful therapeutic management offer a practical reference for both identifying analogous infections and guiding antibiotic selection.

Several limitations must be acknowledged. As a single case report, the findings cannot be generalized to all patients with VP shunt infections. The clinical presentation and treatment response may vary across different patient populations. Retrospective data collection may have introduced bias. Furthermore, while the 10-month follow-up is substantial, it remains insufficient to assess very late recurrence or long-term neurological sequelae that may emerge beyond this period.

## 4 Conclusion and recommendations

In this case, the infected shunt was removed and replaced with sequential external ventricular and lumbar cistern drainage. Targeted antimicrobial therapy, guided by drug susceptibility testing, resolved the CNS infection. Follow-up revealed no recurrence or related complications. This standardized protocol proved safe and effective, providing a valuable reference for managing rare multidrug-resistant infections following VP shunt surgery.

Fever is the most common symptom of CNS shunt infection, and patients with fever after VP shunt surgery should always be suspected of having shunt infection, and the shunt tube can serve as a route for retrograde infection of intestinal bacteria to the CNS.

## Data Availability

The original contributions presented in the study are included in the article/supplementary material, further inquiries can be directed to the corresponding author.
